# Supplementary material for: Opiate Prescriptions Vary among Common Urologic Procedures: A Claims Dataset Analysis
Source: J Clin Med. 2022 Feb 28;11(5):1329. doi: 10.3390/jcm11051329 (PMC8911322; doi:10.3390/jcm11051329)
Supplement: Supplementary file 1 [file jcm-11-01329-s001.zip › jcm-1604482-supplementary.pdf]

Supplemental Table S1. CPT codes for procedures analyzed in this study.

Procedure	CPT Code	Type of Surgical Procedure
Posterior urethroplasty	53415	Reconstructive
Anterior urethroplasty	53410	Reconstructive
First stage urethroplasty	53400	Reconstructive
Second stage urethroplasty	53405	Reconstructive
First stage hypospadias	54304	Reconstructive
Second stage hypospadias < 3 cm	54308	Reconstructive
Hypospadias cripple	54352	Reconstructive
Urethrectomy (male)	53215	Reconstructive
Closure of urethrocutaneous fistula	53520	Reconstructive
Rectourethral fistula repair	45820	Reconstructive
Perineal prostatectomy, subtotal	55801	Major oncologic—pelvic
Tunica vaginalis flap/adjacent tissue transfer (<10 cm)	14040	Reconstructive
Buccal mucosa harvest (full thickness graft)	15240	Reconstructive
Cystoscopy, clot evacuation	52001	Transurethral bladder
Transurethral resection of prostate	52601	Transurethral bladder
Transurethral resection regrowth	52630	Transurethral bladder
UroLift	52441	Transurethral bladder
Additional UroLift	52442	Transurethral bladder
High power potassium-titanyl phosphate laser of prostate	52647	Transurethral bladder
Holmium laser enucleation of prostate	52649	Transurethral bladder
Transurethral incisions of bladder neck contracture	52640	Transurethral bladder
Prostate needle biopsy	55700	Transurethral bladder
Transrectal ultrasound guidance	76942	Transurethral bladder
Cystolithalopaxy (<2.5 cm)	52317	Transurethral bladder
Cystolithalopaxy (>2.5 cm)	52318	Transurethral bladder
Ureteroscopy with stone extraction	52352	Stone
Ureteroscopy with laser lithotripsy (no stent)	52353	Stone
Ureteroscopy with laser lithotripsy (with stent)	52356	Stone
Percutaneous nephrolithotomy < 2 cm	50080	Stone
Percutaneous nephrolithotomy > 2 cm	50081	Stone
Renal endoscopy with stone removal	50561	Stone
Shockwave lithotripsy	50590	Stone
Cystoscopy with biopsy	52204	Transurethral bladder

Cystoscopy with urethral dilation	52281	Transurethral bladder
Direct vision internal urethrotomy	52276	Transurethral bladder
Cystoscopy, external sphincterotomy	52277	Transurethral bladder
Transurethral resection of bladder tumor < 0.5 cm	52214	Transurethral bladder
Transurethral resection of bladder tumor 0.5–2.0 cm	52234	Transurethral bladder
Transurethral resection of bladder tumor 2.0–5.0 cm	52235	Transurethral bladder
Transurethral resection of bladder tumor > 5 cm	52240	Transurethral bladder
Cystoscopy and stent removal (simple)	52310	Transurethral bladder
Cystoscopy and stent removal (complex)	52315	Transurethral bladder
Cystoscopy with ureteral catherization	52005	Transurethral bladder
Cystoscopy with ureteral stent placement	52332	Transurethral bladder
Ureteroscopy, diagnostic	52351	Stone
Ureteroscopy treat ureteropelvic junction stricture	52345	Stone
Ureteroscopy treat ureteral stricture	52344	Stone
Ureteroscopy with biopsy	52354	Stone
Ureteroscopy with resection of tumor	52355	Stone
Renal endoscopy, diagnostic	50551	Stone
Renal endoscopy with biopsy/fulguration/resection	50576	Stone
Percutaneous access for nephroscopy if no tube left	50395	Stone
Placement of percutaneous nephrostomy set after nephroscopy (including initial percutaneous access)	50432	Stone
Placement of nephroureteral stent after nephroscopy (including initial perc access)	50433	Stone
Convert percutaneous nephrostomy set to nephroureteral stent	50434	Stone
Exchange percutaneous nephrostomy set (not initial)	50435	Stone
Place antegrade stent after nephroscopy (not initial)	50693	Stone
Place only antegrade stent after nephroscopy (not initial)	50694	Stone
Place antegrade stent, with separate percutaneous nephrostomy set (not initial access)	50695	Stone
Partial cystectomy (simple)	51550	Major oncologic—pelvic
Cystectomy	51570	Major oncologic—pelvic
Cystectomy ileal conduit	51590	Major oncologic—pelvic
Cystectomy ileal conduit and pelvic lymph node dissection	51595	Major oncologic—pelvic
Cystectomy neobladder	51596	Major oncologic—pelvic
Ureterotomy stent	50605-50	Major oncologic—kidney

Ileal conduit	50820-50	Major oncologic—pelvic
Neobladder	50825	Major oncologic—pelvic
Pelvic lymph node dissection	38770	Major oncologic—pelvic
Radical retropubic prostatectomy	55840	Major oncologic—pelvic
Radical retropubic prostatectomy and pelvic lymph node dissection	55845	Major oncologic—pelvic
Open nephrectomy (includes nodes and inferior vena cava thrombus)	50230	Major oncologic—pelvic
Open partial nephrectomy	50240	Major oncologic—pelvic
Retroperitoneal lymph node dissection	38780	Major oncologic—pelvic
Simple retropubic prostatectomy	55831	Major oncologic—pelvic
Simple suprapubic prostatectomy	55821	Major oncologic—pelvic
Revision of urostomy (to skin)	50727	Reconstructive
Ureteroplasty	50700	Reconstructive
Ureteroneocystotomy (single)	50780	Reconstructive
Ureteroneocystostomy with hitch or flap	50785	Reconstructive
Partial cystectomy and ureteral reimplant	51565	Reconstructive
Ureterolysis for retroperitoneal fibrosis	50715	Reconstructive
Ureteroureterostomy	50760	Reconstructive
Ureterocalycostomy	50750	Reconstructive
Ureteroenterostomy	50800	Reconstructive
Ileal ureter	50840	Reconstructive
Excision of penile plaque with graft up to 5 cm	54111	Reconstructive
Laparoscopic/robotic cyst decortication	50541	Major oncologic—kidney
Laparoscopic/robotic renal cryoablation	50542	Major oncologic—kidney
Laparoscopic/robotic simple nephrectomy	50546	Major oncologic—kidney
Laparoscopic/robotic radical nephrectomy	50545	Major oncologic—kidney
Laparoscopic/robotic nephroureterectomy	50548	Major oncologic—kidney
Laparoscopic/robotic partial nephrectomy	50543	Major oncologic—kidney
Laparoscopic/robotic radical prostatectomy	55866	Major oncologic—kidney
Laparoscopic/robotic lymph node dissection	38571	Major oncologic—pelvic
Laparoscopic/robotic pyeloplasty	50544	Reconstructive
Laparoscopic/robotic adrenalectomy	60650	Major oncologic—kidney
Prosthetics		Prosthetic
Inflatable penile prosthesis	54405	Prosthetic

Artificial urinary sphincter	53445	Prosthetic
Malleable penile prosthesis	54400	Prosthetic
Inflatable penile prosthesis revision	54408	Prosthetic
Remove/replace inflatable penile prosthesis	54410	Prosthetic
Female sling for incontinence	57288	Prosthetic
Male sling for incontinence	53440	Prosthetic
Artificial urinary sphincter revision/removal	53447	Prosthetic
Penectomy	54125	Inguinal/scrotal
Partial penectomy	54120	Inguinal/scrotal
Spermatocelectomy	54840	Inguinal/scrotal
Hydrocelectomy (unilateral)	55040	Inguinal/scrotal
Hydrocelectomy (bilateral)	55041	Inguinal/scrotal
Hydrocele repair bottle type	55060	Inguinal/scrotal
Varicocelectomy	55530	Inguinal/scrotal
Vasovasostomy	55400	Inguinal/scrotal
Vasoepididymostomy (unilateral)	54900	Inguinal/scrotal
Biopsy of testis, incisional	54505	Inguinal/scrotal
Scrotal orchiectomy	54520	Inguinal/scrotal
Inguinal (radical) orchiectomy	54530	Inguinal/scrotal
Reduction in torsion and contralateral fixation	54600	Inguinal/scrotal
Excision appendix testis	54830	Inguinal/scrotal
Excision of spermatic cord lesion	55520	Inguinal/scrotal
Circumcision	54161	Inguinal/scrotal

Supplemental Table S2. List of opioid and opioid-like medications analyzed in this study.

**Opioid only**	Morphine<br>Hydromorphone<br>Fentanyl<br>Methadone<br>Codeine<br>Hydrocodone<br>Buprenorphine<br>Piritramide<br>Meperidine<br>Pentazocine<br>Butorphanol<br>Oxymorphone<br>Nalbuphine<br>Oxycodone
**Opioid with acetaminophen**	Hydrocodone with acetaminophen<br>Oxycodone with acetaminophen<br>Codeine with acetaminophen
**Opioid-like**	Tramadol<br>Tapentadol
